# Forecast Hesitancy: Why are People Reluctant to Believe, Accept, or Respond to Various Weather, Water, and Climate Hazard-Related Forecasts?

**DOI:** 10.1007/s13753-021-00353-7

**Published:** 2021-05-26

**Authors:** Michael H. Glantz, Gregory E. Pierce

**Affiliations:** Consortium for Capacity Building (CCB), Institute for Arctic and Alpine Research (INSTAAR), University of Colorado, Boulder, CO 80301 USA

**Keywords:** Disaster risk reduction, Forecast, Forecast hesitancy, Hesitancy, Hydromet hazards, Vaccine hesitancy

## Abstract

Current discussions of the social phenomenon of “vaccine hesitancy” with regard to Covid-19 provide an opportunity to use hesitancy as a means to shift thinking about untimely and delayed responses to forecasts of hydrometeorological hazards. Hesitancy, that is, provides a paradigm through which such regrettably delayed responses to hydromet hazards might be better understood and effectively addressed. Without exaggeration, just about every hydromet event provides an example of how hesitancy hinders individual, community, and national government risk-reducing preventive and mitigative responses to forecasts of foreseeable, relatively near-term climate, water, or weather hazards. Reasons for such hesitancy (for vaccine and forecast use alike) include—among others—lack of trust in the science, lack of confidence in government, and persistent concern about the uncertainties that surround forecasting—both meteorological and public health. As such, a better understanding of the causes that lead to individual and group hesitancy can better inform hydromet forecasters and affected communities about ways in which beneficial actions in response to timely forecasts are often delayed. This better understanding will facilitate, where necessary, targeted interventions to enhance the societal value of forecasting by reducing this long-observed challenge of “forecast hesitancy.” First, this article focuses on incidents of “vaccine hesitancy” that, for various reasons, people around the world are even now experiencing with regard to several now-available, and confirmed efficacious, Covid-19 vaccines. Reports of such incidents of indecisiveness first increased dramatically over the first few months of 2021, despite the strong scientific confidence that vaccination would significantly lower personal risk of contracting as well as spreading the virus. After, the notion of forecast hesitancy with regard to hydrometeorological hazards is discussed.

*It’s not what you say, it’s what people hear*.

-Frank Luntz (2007)

## Introduction

Following the World Health Organization’s official declaration of the Covid-19 pandemic in mid-March 2020, several national governments and pharmaceutical firms competitively embarked on accelerated efforts to develop a new vaccine to protect populations from the debilitating if not deadly effects of SARS-CoV-2, the zoonotic virus first identified in Wuhan, China in late 2019 that causes the pandemic disease. By the end of 2020, a number of these efforts proved successful. Accelerated efforts at research, development, and clinical trials produced several efficacious vaccines. The record-setting pace of Covid-19 vaccine development is a game-changing step toward ending the pandemic. It will contribute to increased seroprevalence across the world, which is key to reaching so-called herd immunity against the most serious effects of viral infection (see Taylor 2021 for a coronavirus pandemic timeline).

Equitable distribution of the vaccines around the globe has, however, been much easier said than done. It has not been without challenges because, among other reasons, the quick development of the vaccines has drawn a lot of attention, especially from people who are skeptical of vaccine safety or the need for vaccination in general. These individual and group concerns have led to some public controversy in most countries around the world, from those in the industrial world who are “vaccine haves” to those in the developing world who are “vaccine have-nots.” This controversy can be summed up in one question that expresses the doubt many people are now feeling: to take the vaccine or not to take the vaccine? Such doubt should really be understood more broadly, however, as representative of the playing out of several other, more wide-ranging questions that are all in essence about hesitancy. These questions hinge on “to do or not to do?”—to trust or not to trust the safety of the vaccine, to trust or not to trust government statements about the vaccine, to trust or not to trust the competency of the vaccine suppliers or givers of the vaccine, and so on.

There are plausible reasons, concerns, and excuses, not to mention matters of mis- as well as dis-information, that anyone can offer to argue against taking advantage of the Covid-19 vaccines now, or in a while, or ever. Likewise, there are many legitimate reasons why people hesitate to take decisive action in response to new forecasts of foreseeable—but not assured—hydrometeorological hazard events. Like this, some degrees of hesitancy can be viewed in a positive light; however, hesitancy tends to suggest a negative response to a timely forecast—in public health or in meteorology—as with the adage, “Those who hesitate are lost.”

An Internet search for reluctance to commit to taking advantage of the vaccines yields countless articles and social media discussions related to “vaccine hesitancy,” which is currently on the minds of the public as well as of public health officials since the increasing availability of the various new Covid-19 vaccines has made large-scale positive reception essential. Aware of this essential public need of positive vaccine reception, at least since the beginning of the last decade the public health community has pointedly sought to identify the reasons behind varying degrees of hesitancy to vaccination, which had been a known problem since at least the introduction of the first smallpox serum in the late eighteenth century.

A more recent article about vaccines, published five years before the outbreak of Covid-19, first drew our attention to this long-perplexing notion of “vaccine hesitancy.” In that particular article that first drew our attention to the notion, MacDonald and The SAGE Working Group on Vaccine Hesitancy (2015, p. 4261; see also SAGE 2014), writing about the World Health Organization’s (WHO) SAGE Working Group on Vaccine Hesitancy, notes:

Vaccine hesitancy refers to delay in acceptance or refusal of vaccination despite availability of vaccination services. Vaccine hesitancy … is influenced by factors such as complacency [those who do not perceive a need for a vaccine, do not value the vaccine], convenience [those who have access issues] and confidence [those with a low level of trust in vaccine or provider].

The Covid-19 pandemic has exposed the general public to the overarching concept of vaccine hesitancy, a social phenomenon that, though worried over by public health professionals since at least the development of a first effective smallpox vaccine in the 1790s, was only formally defined within the past 10 years when scientists were called upon by WHO to define the phenomenon. As a result, the Strategic Advisory Group of Experts (SAGE) Working Group on Vaccine Hesitancy was convened in 2012. Two years later, SAGE (2014) published its definition.

As the history of vaccines shows, announcement of the development of any new vaccine to curtail the effects of an emerging viral threat can be expected to lead to responses from the public that vary along a continuum. This continuum will range from the positive extreme, a total belief in and acceptance of a new vaccine, to the negative extreme, which entails an outright rejection of the new vaccine. Schuster et al. (2015, p. 4159) support this interpretation, observing:

The recognition that vaccine hesitancy is complex with many different determinants that vary with context, vaccine, setting and time infers that it is unlikely that any single strategy would be effective in addressing all determinants of vaccine hesitancy.

A review of recent surveys in the United States shows peaks and troughs and peaks again among those who would take the vaccine immediately upon availability and those who would take it only after it had been tested by others over some period of time to be assured there would be no negative side effects. Still others refused to take the vaccine under any condition. Members of each group had their specific reasons.

Many surveys about vaccine hesitancy have at this point been conducted at scales ranging from the global to the local. Responses to one such survey (News-medical 2021), for example, found:

The top four reasons given for vaccination hesitancy were as follows: concerns about vaccine side effects, worries about allergic responses to the vaccine, doubts about vaccine effectiveness and a preference for developing immunity through infection. Other reasons were less frequently cited—including being healthy, fear of needles, being immune from past infection, being young and lack of concern about developing a serious illness.

Other recent, peer-reviewed surveys, such as that conducted by Paul et al. (2020), have made similar findings.

Although the term “vaccine hesitancy” has only relatively recently been formally defined as an overarching concept that captures various experiences of reluctance among different publics to accept an efficacious vaccine, the onset of the Covid-19 pandemic has led to a definite expansion of the scientific literature as well as of media representations of the phenomenon. This attention affirms how viruses pose a clear and, today, particularly present hazard to individuals and communities from the time of an outbreak until a vaccine has been developed and mass distributions of efficacious “shots in the arm” have been undertaken worldwide.

In comparing concerns about generating timely responses to a vaccination campaign in the face of an emerging epidemic to concerns about generating timely responses to a forecast of an emerging, potentially high impact El Niño event, for example, the question arises: Could the notion of “vaccine hesitancy” add value to present understandings of the differing societal responses to hydrometeorological forecasts? We feel that the answer is definitely “yes”! Evaluating “forecast hesitancy” as a useful paradigm to increase understanding of delayed responses to forecasts would be beneficial to a number of fields, including meteorological science and societal impacts research.

## Defining Forecast Hesitancy

Individuals and groups frequently hesitate to act on forecasts as early warning of potential harm until their understandings, or beliefs, change about the uncertainties of the imminence of the probable threat (Taylor et al. 2015). Hesitancy has like this played out consistently for a wide range of hydromet forecast situations over the last many decades. Despite the recurring incidence of forecast hesitancy worldwide, however, the disaster risk reduction (DRR) community, collectively, has yet to develop strategic or tactical criteria for response that might reduce the challenges to forecast effectiveness posed by delayed responses to hydromet hazard forecasts.

Having recognized several parallels between efficacious vaccines and hydromet forecasts, we believe hesitancy, as an emotional response to probabilistic science uncertainty, might provide a useful paradigm through which current understandings of this often-observed phenomenon of “forecast hesitancy” can be further defined, clarified, and deepened. As such, it offers a fresh perspective on the phenomenon, one that might enable the development of solutions that reduce vulnerability within populations ever living in the shadow of the next hydromet hazard event.

Identifying reasons for delayed responses could uncover lessons to be learned from each of the two distinctive hesitancies. Research, lessons identified, lessons learned, and responses to the use, or lack thereof, of a protective vaccine for a dangerous new threatening virus can provide new insights into why individuals, communities, and governments so often unwittingly choose to hesitate to respond in a timely manner to forecasts of extreme hydromet events.

A working definition of “forecast hesitancy,” therefore, is as follows: “The varying degrees of reluctance by different individuals, groups, communities, and nations to respond to or rely on forecasts in order to take advantage of forecast-afforded lead time to prepare effectively for the threats that are known to accompany different hydromet hazards.”

Such hydromet hazards can include floods, flash floods, tornadoes, heatwaves, ice storms, tropical storms, forest and brush fires, dry spells and droughts, disease outbreaks, and ENSO-extreme anomalies of El Niño and La Niña.

The point is that people tend to be overburdened by competing and often conflicting government and private forecasts of such hazards. These forecasts typically appear in print and electronic media, including on social networks (Freedman 2019). Daily weather forecasts are probably the most frequently issued, though they likely have the least consequential impacts. Nevertheless, such forecasts tend to be sought after daily, sometimes more actively than others. Forecasts of severe hydromet hazards, though less frequent, tend to be more consequential in terms of the well-being of individuals, communities, and governments at all levels. Such hazards can be life and livelihood threatening as well as destructive of natural environments and built infrastructures.

Forecasts of such extremes are mostly heard but are not always heeded. The reason is that though forecasts are in essence early warnings of potential disasters, they may not be viewed as such. Because they often occur in a series, as with advisories, alerts, warnings, and watches, which correspond to increased certainty of a hazard event, many people mistakenly believe such early forecast products are merely informational. They often do not register such products as providing them with a direct, consequential service. They see them more as just another form of news, to be heard with some interest perhaps but not really to be heeded right away.

What many people do not realize is just how critical understandings of such forecast warnings are. But most people also do not realize that the earliest possible warning is their own knowledge of their specific home region’s climatology—especially its seasonality and its extremes. With hindsight, however, each type of hydromet hazard that has become a disaster has its own set of stories of preventable losses of life, livelihood, and property. Preventable in this case means that had early warnings been both heard and heeded by at-risk populations, some portion of the losses in any hazard event would likely have been avoided.

Again, most people do not realize that forecasts are always originally given in probabilistic terms. Statisticians know well how to view and evaluate the meaning of such probabilistic forecasts, but the public in general may not be able to process this information in the same way. As a result, losses often occur when individuals and communities are confronted by a known-to-the-region hydromet hazard the solid technical forecast warning for which was not well-communicated to the public, even though a number of detailed forecast alerts with plenty of lead time might have been issued. A warning is just noise that overshadows the signal—the dire warning of impending impacts—if it is not understandable to the audience intended.

## A Texas Case Study: The 2021 Polar Vortex

Recent events in the state of Texas provide a profoundly illustrative (and very disturbing) example of the negative impact on society of government officials’ hesitancy in responding to, in this case, a monthlong series of hydromet hazard forecasts. In mid-February 2021, residents and officials across the normally temperate southern U.S. state were “surprised” by, and quite ill-prepared for, the onset of consecutive days of record-shattering freezing cold. Frigid temperatures across the state dropped below freezing even in places like Houston where February lows are seldom recorded below a cool but comfortable 9°C (48°F) degrees. Non-weatherized equipment across the state’s electrical and natural gas infrastructures froze, forcing Texans residing in all manner of poorly insulated residences that had been designed in conformity to temperate climate expectations to endure 3–8 days of bitter cold. To generate heat, many people were forced to burn their furniture, and over 100 individual deaths have been directly attributed to the extreme conditions. As Srikanth (2021) reports:

Millions were without power and heat for days during historic snowfall and record-low temperatures, and the state’s health department said most of the 111 deaths reported between Feb. 11 and March 5 were associated with hypothermia. Other deaths were caused by motor vehicle accidents, carbon monoxide poisoning, medical equipment failure, exacerbation of chronic illness, lack of home oxygen, falls and fire.

One example of the devastating impacts of the recent disaster is the tragedy of Cristian Pavon Pineda, an 11-year-old boy who perished of hypothermia even as he huddled under several blankets in his family’s home. Many succumbed similarly to the cold, while numerous others died because their life-saving electronic devices were left powerless during the crisis (Salcedo 2021). Also lacking proper weatherization, water pipes in homes, hospitals, businesses, and other municipal facilities burst, leaving millions of people across the state without clean water for drinking, washing, or cooking for days or, in some of the most vulnerable poor and minority communities, for weeks on end.

Although the total cost of the freeze is still being calculated, and also heatedly debated, one recent estimate put total losses at upwards of USD 155 billion (Puleo 2021), which would place the event among the most costly weather-related disasters in U.S. history. As might be expected, finger pointing and deflection especially from majority Texas Republican state officials including Governor Greg Abbott, who quixotically tried to blame energy-producing windmills for the utter failure of power and leadership over the course of the crisis (K. Shepherd 2021), were rampant in what remains even now, months later, a very active blame game.

Overall response to the event in Texas merits characterization as having been no less than an utter failure. It is so classifiable because of the great suffering that followed on after poor planning and response left the state unprepared for just such an event. It is also so classifiable because of Texas’s marked hesitancy—among government officials and to a lesser extent, private citizens who rely on their leaders for guidance—to act on a solid month’s lead time of technically well-forecasted weather information. Deeming the impacts of the polar vortex event that waylaid Texas in mid-February 2021 inevitable, therefore, suggests that it was one of those “surprising” events that are unfortunate but inescapable. This is a patently inaccurate description; the crisis was foreseeable from a mile, and at least a month, away.

Of particular interest in emphasizing the foreseeable nature of the recent Texas polar disaster event is how, as Table 1 shows, by the beginning of the second week of January, over a month before temperatures actually dropped below freezing across Texas, some local newscasts had already begun to run what might be thought of as “first warning” stories about the likelihood of an impending bitter cold event. These stories, such as the one from NBC affiliate KXAN in Austin, often focused on viewer-friendly descriptions of the technical nature of the polar vortex phenomenon as well as on the likelihood that the coming event would cause “volatile” winter weather (Chow 2020).

**Table 1:** Advanced warnings of impending freezing temperatures in Texas from (predominantly) local weather media (8 January to 12 February 2021) (compiled by R.J. Ross)

1st Warning - “Polar vortex may split, causing volatile winter weather locally” (8 January 2021)
https://www.kxan.com/weather/weather-blog/polar-vortex-may-split-causing-volatile-winter-weather-locally/
2nd Warning - “Bitter Arctic outbreak to deliver icy blast to northern U.S., shift southeast” (2 February 2021) https://www.washingtonpost.com/weather/2021/02/02/arctic-outbreak-cold-blast/
3rd Warning - “FIRST WARNING: Extended Arctic blast coming to Texas” (4 February 2021) https://www.kxan.com/weather/weather-blog/first-warning-extended-arctic-blast-coming-to-texas/
4th Warning - “Arctic blast timeline: A Winter Storm Warning for north, NW Harris County will go into effect Saturday night” (10 February 2021) https://www.khou.com/article/weather/Houston-texas-weather-arctic-blast-cold-front-brings-freezing-temps-ice-and-snow/285-a5323959-fc67-4ab0-8d56-89fc1ba7e636
5th Warning - “Get ready for cold: What to know about the coming Arctic blast” (10 February 2021) https://www.click2houston.com/weather/2021/02/10/what-to-know-about-this-arctic-blast/
6th Warning - “Photos: Arctic blast blankets parts of Texas in ice” (12 February 2021) https://www.houstonchronicle.com/news/houston-weather/article/Texas-ice-snow-Houston-arctic-blast-weather-photos-15945806.php

**Fig. 1:**
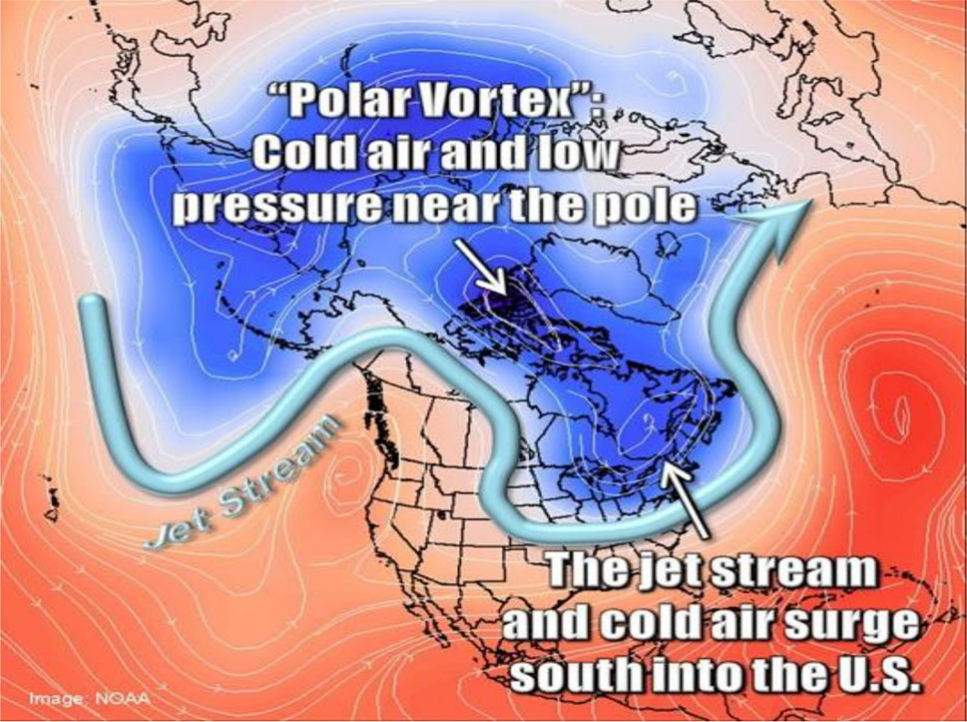
The collapsing polar vortex. *Source* NOAA (2021)

**Fig. 2:**
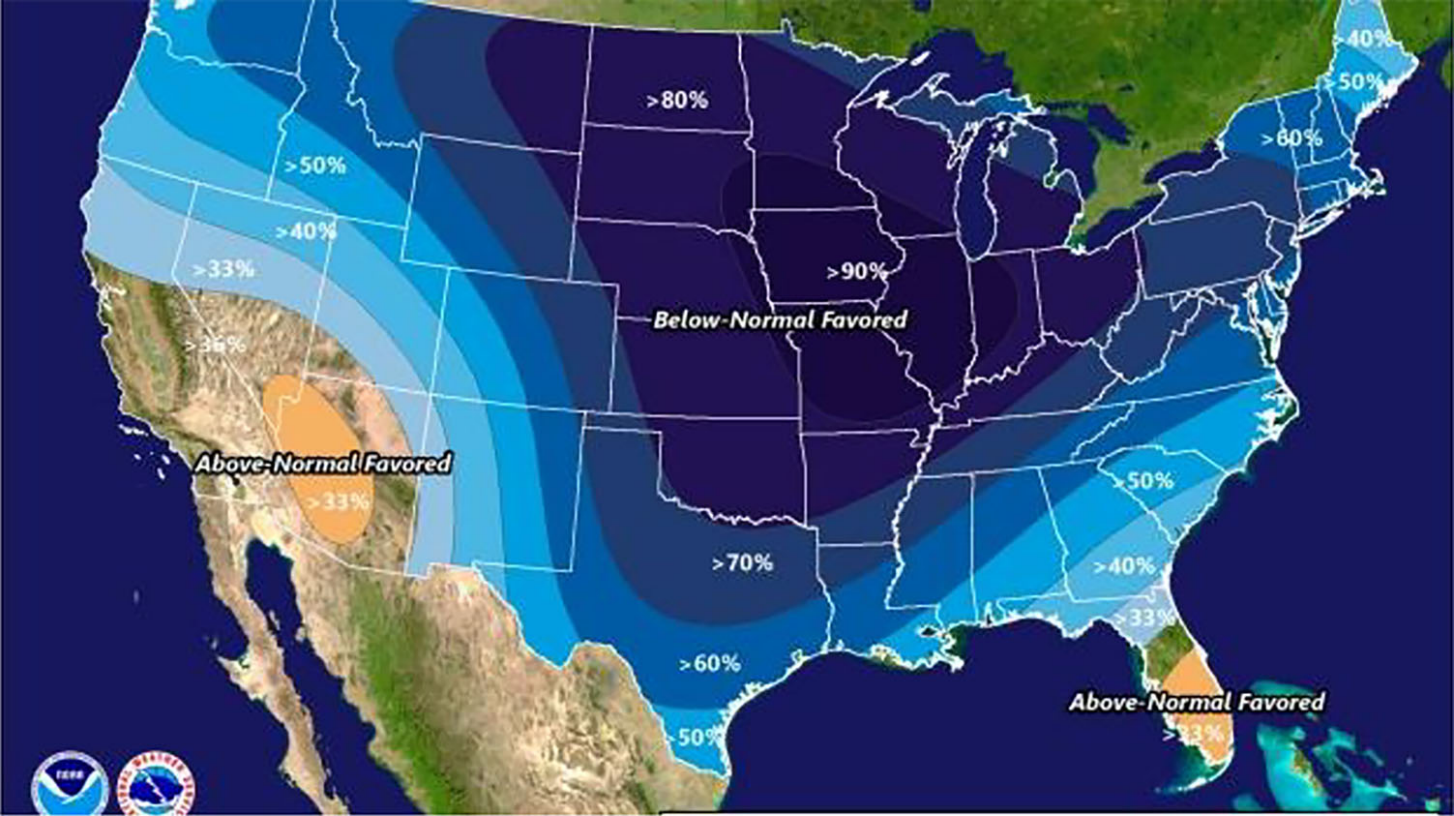
Weather forecast of below-normal temperatures across large parts of the United States *Source* AP (2021)

Accompanying these mid-January first forecast warnings of the coming event were graphics meant to provide further explanation, through illustration, of the destabilizing winds over the circumpolar region (Figs. 1 and 2). At that time, local TV broadcasts daily showed these graphics in their weather updates.

The latter three (4th–6th) early warning entries in Table 1 tell the unfortunate tale of what eventually happened, despite several weeks of lead time from forecasted warnings, over the course of the recent tragedy in Texas. For despite the warnings produced from technical forecasts from the federally run National Weather Service (NWS) and the strong trust that might have been developed considering the substantial lead times those early forecasts had afforded, many individuals across the state still hesitated to respond effectively to the solid, and later confirmed quite accurate, technical information about the coming calamity.

This hesitancy of response was especially aggravated by the politics of emphatically “anti-expert” Texas state government officials who have been especially doubtful of the role of the federal government in its state affairs. Their political “commitments” (really ideology) foreclosed their abilities to act in the best interests of their constituents, thus impeding their respective, and collective, capacities to fittingly heed the warnings of impending disaster they were hearing from technical experts. As a National Weather Service spokesperson (AP 2021) lamented after the event:

The mid-February killer freeze was no surprise and yet catastrophe happened. Meteorologists, government and private, saw it coming, some nearly three weeks in advance. They started sounding warnings two weeks in advance. They talked to officials. They tweeted and used other social media and were downright blunt.

What had long been predicted to happen—and what did happen—is that a low-pressure mass of the sub-freezing air that always exists in the circumpolar region but that is typically locked up in a vigorous counterclockwise wind pattern tightly circling the north pole became unstable and drew down deep across a large swathe of the southern latitudes of the North American continent. This atmospheric anomaly, a hazard referred to by forecasters and in popular media alike as a “polar vortex,” is apparently becoming more frequent as greenhouse gases warm the upper atmosphere (M. Shepherd 2021) and push denser air further into the polar north, destabilizing those vigorous, otherwise steady circumpolar winds in a way that cause them to draw freezing air down as far as the U.S. Gulf Coast (NWS 2021).

The incidence of this polar phenomenon has been known for decades, which means, among other things, that the February 2021 freezing-air crisis in Texas was utterly foreseeable—not only through the technical haze of meteorological probability (and over a century of such known freezes) but also because a similar major event had occurred as recently as 2011. Regrettably, that is, just 10 years prior to the recent tragic events in Texas another dangerous, low-temperature vortex similarly resulted in foreseeable crisis across the state (NWS 2011). The weather crisis back then was also met with surprise, finger pointing, and deflection. It amounted to a similar utter failure of political power and energy infrastructure, an abdication of leadership almost identical to that that was observed—and experienced by the people of Texas—in 2021.

Following on from that 2011 event, as will likely be the case in the coming months following the recent 2021 event, came numerous reports that included specific lessons that were to have been learned. These lessons, as is typical, took the form of specific recommendations on how to prepare for the next polar vortex hazard event. As so often happens (Glantz and Baudoin 2014), however, the lessons outlined in those reports from 2011 were only identified but not truly learned, which essentially means that the tragic outcomes of the 2021 event proved to be a replay of the 2011 event, though with even more devastating impacts in terms of loss of life and property. The 2011 Texas deep-freeze crisis was in retrospect little more than a dress rehearsal for the tragic events of 2021.

As anticipated, recommendations from various sources have already begun to pour in suggesting various solutions for how to protect the state’s energy systems during those future polar vortex events that everyone—meteorologists, government officials, the general public, and so on—knows (or should know) are coming. But, predictably, these recommendations are comprised almost exclusively of narrowly focused technical proposals to yet again review and yet again renovate the state’s energy infrastructure (Layke et al. 2021), which is exactly what the many reports published after the 2011 deep-freeze event had recommended. As America’s Major League baseball great Yogi Berra once memorably said, “It’s like *déjà vu* all over again.”

In addition to the no doubt necessary technical interventions again recommended in reports on the 2021 event are interventions that will contend with the more socio-structural issues that can be understood collectively, we propose, through the paradigm of hesitancy. These are issue having to do with responsive governance, societal expectation, and individual trust—all those various, interacting levels of exchange, perception, and orientation that, when poorly functioning or out of synch, tend to inhibit effective action in response to even the best technical forecasts of impending hydromet hazard impacts.

## Forecast Hesitancy Revisited

In examining the parallels between public health and meteorology in terms of forecasting and outreach, we have found that the SAGE Working Group’s three Cs that factor into vaccine hesitancy—Confidence, Complacency, and Convenience—can also be used effectively to classify the factors of forecast hesitancy. These factors can be used to better understand why people around the world often hesitate to act after they have received technical forecasts of foreseeably destructive impending hydromet extreme events. Table 2 uses El Niño as an example of how SAGE’s three Cs can be usefully adapted to better understand the factors that lead to forecast hesitancy.

**Table 2:** Adaptation of “Forecast Hesitancy” as compared with SAGE’s (2014) three Cs that factor into “Virus Hesitancy”

Application of Three Cs to Forecast Hesitancy	Application of Three Cs to Virus Hesitancy
**Confidence [or Trust, in self or others]**	**Confidence [Trust] in self or others**
Lack of trust in science (*can’t forecast intensity*)	Lack of trust in vaccine safety (*side effects*, *allergies*)
Lack of trust in the government (*Trump’s “SharpieGate”*)	Lack of trust in vaccine effectiveness and a preference
Lack of trust in the forecasters (*tell gov. before public*)	Lack of trust in introduced vs. acquired immunity
Lack of trust in experts (*previous erroneous forecasts*)	Lack of trust in government, drug corporations, data
	Lack of trust in the severity of the threat
**Complacency [perceived risk]**	**Complacency [perceived risk]**
El Niño, for example, involves a series of forecasts as early warning; it is a creeping hydromet hazard	Creates risk by increasing tendency to do nothing or to rely on top-down process for protection
Not conducive to personalities that tend to be . . .	Not conducive to personalities that tend to be . . .
Risk-taking	Unconcerned about health because healthy
Reliant on government (“protect us”)	Fearful of needles
Discounting of lessons from previous events	Certain of acquired immunity from past infection
	Unconcerned about serious illness (due to youth)
**Convenience [access]**	**Convenience [access]**
Communication ranges from good to poor	Access to vaccine
Most at-risk places/people often isolated from constant media reporting	Distance to travel for vaccination
Timing of forecast(s) and remaining lead time from the last of the early warnings can be problematic	Access to transportation

The Strategic Advisory Group of Experts’ original classification of the three Cs was later expanded by Betsch et al. (2018), whose research objective was to develop “a more comprehensive and relevant validated measure of these 5C psychological antecedents of vaccination” (Betsch et al. 2018, p. 1). To that end, they pointed out how structural and psychological barriers also factor into the recurrence of the phenomenon of vaccine hesitancy, proposing two additional Cs (hence, 5Cs) that they argue also play a significant role in explaining hesitancy when it comes to vaccination. Betsch et al.’s additional factors are “Calculation” (engagement in extensive information searching) and “Collective Responsibility” (willingness to protect others).

Evident from these additional factors is how several of the same key concerns exist with regard to hydromet hazard forecasts. Clearly, for example, “trust” is a major factor influencing whether or not timely action will be taken after a forecast has been received. As with vaccines, this includes trust in government officials, in scientific data and in the scientists assessing that data, in transparency, and in the efficacy of response plans and preparations. It also includes equity of information sharing and resources allocation to all groups regardless of race, gender, age, culture, religion, or economic status. In this way, just how an individual or a community “perceives” the risks of a foreseeable virus or impending hazard event affects whether, when, or how that individual or community will respond to calls to prepare for the threats that both past experiences and technical data show they will face. “Access” to efficacious vaccinations or adequate pre-disaster response resources is also a concern, especially when the question arises of whether or not individuals or communities have adequate capacity to utilize effectually those allocated interventions and other resources.

Notable, therefore, is that although trust or confidence is often mentioned as an issue with regard to hesitancy, “lack of trust” or “lack of confidence” has, pointedly, often proven to be the major obstacle to vaccine use. This obstacle, as the Texas case study above illuminates, can be shown to parallel how hesitancy often hinders action in response to even the best technical forecasts of impending climate, water, and weather hazard events.

Identifying parallel concerns having to do with timely positive responses to a vaccination campaign during an ongoing epidemic to those having to do with timely responses to forecasts of an impending high-impact hydromet event suggests that the notion of “hesitancy” adds value to present understandings of the differences between various societal responses to hydromet forecasts. Identifying these parallels will improve current understandings of this long-recognized challenge of what we propose here as “forecast hesitancy.”

As noted above, for example, with the announcement of any new vaccine for a novel virus, responses from the public are sure to vary along a continuum from total belief to total rejection. Mezuk (2019) has shown that responses to forecasts of potentially high-impact climate and weather hazard events tend to vary along a similar continuum. Identification of this continuum of hesitancy shows that a universally accepted response that addresses the range of sometimes conflicting reasons for why different people hesitate to act in the face of impending hazards is not to be expected. Hesitancy to act effectively in response to hydromet hazards is, as Schuster et al. (2015) observed with regard to vaccine hesitancy, subject to the same range of social complexities and determinants that make unlikely a single strategy for adequate overall response.

One such complexity, for example, is the range of temporal onsets associated with different types of hydromet hazards. Each type of event has its own set of forecasts related to its rate of onset and those ranges vary—from minutes (tornadoes, flashfloods) to months (drought, floods) and even to years (hurricanes/cyclones/typhoons, El Niños). Timely effective action in response to the risk posed by any one of these hazards requires ample lead time that enables different populations to decide when and how they will act. This requirement only increases the amount of overall complexity involved in responding to even a single hazard event. The technical capacity now available for forecasting such events is rather astounding. Despite this capacity, however, many people, especially those who fall to the rejection side of the hesitancy continuum, remain reluctant to act even as forecasts become increasingly certain as the time of impact draws nearer. Such a hesitant response provides an excellent example of how merely issuing a solid technical forecast is not enough—people often hear without adequately heeding the warnings, thus adding to their own vulnerability to a hazard the impact of which need not end in tragedy.

Timeliness, in this way, is crucial to mitigation of hazard risk. Timeliness in forecasting specifically refers to the lead time provided to citizens that enables them to ready themselves strategically or tactically for what adverse, potentially life-threatening impacts they should foreseeably expect with the onset of a specific hazard. Table 3 charts the lead times typically available between first forecasts (as warnings) and the first sign of onset of impacts for different hydromet hazards.

**Table 3:** Different hazards and suggested lead times as estimated periods between first warning and impact onset

Type of Hydromet Hazard	Time of Occurrence	Quick or Slow Onset	Time between First Warning and Impact
Flood	Anytime	Slow	Days, weeks
Flash Flood	Anytime	Quick	Minutes, hours, day(s)
Severe Storm	Seasonal	Quick	Minutes, hours, day(s)
Tornado	Seasonal	Quick	Season
Blizzard	Seasonal	Slow	Days, week(s)
Ice Storm	Seasonal	Slow	Hours, days
Hurricane	Seasonal	Slow	Years (climatology), season, weeks, days, hours
Typhoon	Seasonal	Slow	Years (climatology), season, weeks, days, hours
Cyclone	Seasonal	Slow	Years (climatology), season, weeks, days, hours
Disease Outbreak	Anytime	Quick	Season, weeks, days
Epidemic	Anytime	Slow	Weeks, days
Pandemic	Anytime	Slow	Months, weeks
Drought	Seasonal	Slow	Months, weeks, days
Dry Spell	Seasonal	Slow	Days, weeks
El Niño / La Niña	Sub-Decadal	Slow	Season, months, weeks

What Table 3 suggests is that the ultimate goal of forecasters is to expand the lead times for alerting people to risks associated with the various hazards so as to improve each warning’s timeliness and, in essence, its usability. Doing so is often already within the realm of possibility for technocrats in those government agencies that are responsible for producing and disseminating forecasts and other warnings.

## Concluding Comments

Developing a paradigm of “forecast hesitancy” will, as with its increasingly well-known public health antecedent vaccine hesitancy, enable elaboration of socio-culturally suitable remedies to those long-known challenges that have continued to elude the promise of improved technical interventions. Problems arise when people, for various reasons typically having to do with lack of trust, complacency, or convenience, hesitate—or outright fail—to act on available science-based forecast information as early warning. Specifically outlined in this article is how a better understanding of the reasons for individual and community vaccine hesitancy can inform hydromet forecasters about different ways to understand, approach, and overcome at least some of the distinctive challenges posed by “forecast hesitancy” for disaster risk response and reduction.

Just as with new technologies that have enabled swifter development of efficacious vaccines for emerging viral threats like Covid-19, so are the forecasting sciences consistently improving in terms of predictive capacity for hydromet hazards. History shows that there have been surprising hydromet disasters that in retrospect should not have been labeled as surprising. Still, there have always been and likely always will be doubters, those who fall to the rejection side of the hesitancy continuum. The persistent tendency of these groups of doubters to emerge with the development of each new vaccine convinced WHO almost a decade ago of the need to better understand why some people are reluctant to immediately seek the advantages of vaccines once they come available and have proven safe and effective. By convening the SAGE Working Group, WHO signaled its interest in addressing the issue not only as a technical problem but also as a social phenomenon.

Comparing concerns about timely responses to a vaccination campaign in the face of an existing epidemic to concerns about timely responses to forecast warnings of an emerging, high-impact hydromet threat suggests that the notion of “hesitancy” can add value to present understandings of the different societal responses to hydromet hazard forecasts as they fall along the hesitancy continuum. The objective of this comparison is to improve current understandings of the long-observed challenge of “forecast hesitancy.”

Would it be beneficial to the community of national meteorological and hydrological services (NMHSs), as represented by WMO (World Meteorological Organization), to follow the example set by WHO for vaccine hesitancy by identifying the range of underlying and precipitant factors that prompt reluctance to treat hydromet hazard forecasts as early warnings? Lead time for people to prepare for such hazards is a valuable commodity. Understanding hesitancy as a phenomenon grounded in emotional responses to impending threats provides a paradigm through which social behaviors can be accurately identified and suitably modified. In this way does “forecast hesitancy” as proposed in this article demand serious consideration and a formal definition.
